# Grip Strength Moderates the Association between Anthropometric and Body Composition Indicators and Liver Fat in Youth with an Excess of Adiposity

**DOI:** 10.3390/jcm7100347

**Published:** 2018-10-12

**Authors:** Robinson Ramírez-Vélez, Mikel Izquierdo, Jorge Enrique Correa-Bautista, Alejandra Tordecilla-Sanders, María Correa-Rodríguez, Jacqueline Schmidt Rio-Valle, Emilio González-Jiménez, Katherine González-Ruíz

**Affiliations:** 1 Centro de Estudios Para la Medición de la Actividad Física CEMA, Escuela de Medicina y Ciencias de la Salud, Universidad del Rosario, Bogotá 111221, Colombia; jorge.correa@urosario.edu.co (J.E.C.-B.); alesanders_0615@hotmail.com (A.T.-S.); katherine.gonzalez@docentes.umb.edu.co (K.G.-R.); 2 Department of Health Sciences, Public University of Navarre, CIBER de Fragilidad y Envejecimiento Saludable (CB16/10/00315), Tudela, Navarre 31006, Spain; mikel.izquierdo@gmail.com; 3 Departamento de Enfermería, Facultad de Ciencias de la Salud, Avda. De la Ilustración, 60, University of Granada, Granada 18071, Spain; macoro@ugr.es (M.C.-R.); jschmidt@ugr.es (J.S.R.-V.); emigoji@ugr.es (E.G.-J.); 4 Grupo de Ejercicio Físico y Deportes, Facultad de Salud, Programa de Fisioterapia, Universidad Manuela Beltrán, Bogotá 110231, Colombia

**Keywords:** handgrip strength, cardiorespiratory fitness, fatty liver, adiposity, youths

## Abstract

Paediatric non-alcoholic fatty liver disease (NAFLD) is considered the most common early driver of chronic liver disease. The aim of this study was to examine whether grip strength moderates the association between anthropometric and body composition parameters and controlled attenuation parameter (CAP), an indicator of fat deposits in the liver, in children and adolescents with excess of adiposity. A total of 127 adolescents (67% girls) aged between 11 and 17, attending two public schools in Bogotá (Colombia), who had an axiological evaluation of obesity were included in this study. A grip strength test was assessed as an indicator of muscular strength, and cardiorespiratory fitness by maximal oxygen uptake was assessed using the 20 m shuttle-run test. Waist circumference (WC), waist-to-height ratio (WHtR), fat mass, and visceral adipose tissue (VAT) (cm^3^) were included as anthropometric and body composition measures. CAP was determined with a FibroScan^®^ 502 Touch device (Echosens, Paris, France). The anthropometric and body composition parameters including WC, WHtR, fat mass, and VAT were positively associated with the CAP (range β = 0.423 to 0.580), slightly reduced after being adjusted for handgrip strength/weight. The Johnson-Neyman technique revealed a significant inverse relationship between WC, WHtR, VAT, and CAP when grip strength normalized by body mass was above but not equal to or below 0.475 (8.1% of the sample), 0.469 (8.9% of the sample), and 0.470 (8.5% of the sample), respectively. In conclusion, grip strength adjusted by body mass, has a moderating effect on the association between anthropometric and body composition parameters (including WC, WHtR, and VAT) and CAP in in children and adolescents with excess of adiposity, suggesting the importance of promoting muscular strength during paediatric population in order to prevent NAFLD.

## 1. Introduction

Paediatric non-alcoholic fatty liver disease (NAFLD) is considered a hepatic manifestation of metabolic alterations and is the most common early driver of chronic liver disease in industrialized and developing countries [1]. It is characterized by fat accumulation, mainly as triglyceride, inside liver cells, and NAFLD may progress through three different stages starting from NAFLD, to non-alcoholic steatohepatitis (NASH), and finally liver cirrhosis [1]. The prevalence of NAFLD varies among ethnic/racial groups [2], with the Latin American population disproportionately affected. The prevalence of NAFLD in obese paediatric populations has been reported to range from 7.6% to 34.2% [3]. It is well established that a sedentary lifestyle increases the risk of obesity and metabolic syndrome. However, recently it has been proposed that sedentary lifestyle could also be responsible for the high prevalence of NAFLD, as it is known to increase in parallel with obesity, metabolic syndrome, type 2 diabetes, and particularly visceral fat obesity, an important clinical target for managing the negative consequences of obesity in children and adolescents [3].

Muscular fitness is a global term that includes the phenotypes or observable characteristics of muscular strength, muscular power, and local muscular endurance [4]. In general, muscular fitness (determined using a grip dynamometer in this study) seems more positively associated with mobility impairment and long-term mortality [5], but is less commonly measured in clinical practice that other physical performance as isotonic or isoinertial methods. Accumulated evidence shows that healthy muscle strength is closely related to improved insulin sensitivity [6], modulated insulin secretion, and ameliorated dyslipidemia [7], the principal risk factors for developing NAFLD [8]. In this context, and considering that up to 25% of South Korean adults patients with NAFLD were not obese [9], factors other than obesity such as unfavourable adipose tissue distribution or low skeletal muscle mass might contribute to NAFLD. Thus, previous studies have found a positive relationship between muscular skeletal mass and CAP, independent of obesity, insulin resistance, or metabolic syndrome in population of adults [10,11]. However, this relationship has not been previously investigated in young population.

The term dynapenia (“dyna” = power and “penia” = deficiency), was proposed to highlight the observable impact of low levels of muscular fitness and consequent functional limitations not caused by neurologic or muscular disease [12]. Previous reports have suggested that paediatric dynapenia is a contemporary corollary of modern-day lifestyles characterized by physical inactivity and a high degree of sedentary behaviour [13,14]. In fact, a previous meta-analysis investigating whether grip strength changed on average over recent decades in children and adolescents versus adults in Canada and the US found that grip strength in children and adolescents are weaker and slower than previous generations [15]. Similar trends have been reported for American young people [16], English children [17], Spanish adolescents [18], and Latin American adolescents [4]. Since muscular strength have emerged as important mechanisms involved in the development and progression of NAFLD, we hypothesized that grip strength might moderate the positive association between anthropometric/body composition indicators and NAFLD in children and adolescents.

To date, most previous studies have investigated the influence of physical exercise on cardiometabolic risk factors including NAFLD, suggesting that this promotes health benefits for NAFLD patients, independently of weight reduction [19,20,21]. Perseghin et al. [21] reported that a higher level of habitual physical activity was associated with a lower intrahepatic fat content. Similarly, in a cross-sectional analysis of 72,359 healthy Korean adults, regular exercise was associated with a reduced risk of contracting NAFLD [9].

In addition, it has been reported that resistance training (RT) specifically improves NAFLD, independently of any change in body weight, demonstrating that a RT program brought about an approximately 13% reduction in intrahepatic lipids [22]. Zelber-Sagi et al. [23] concluded that RT may complement NAFLD treatment as it improves hepatic fat content. Likewise, a recent systematic review and meta-analysis showed that supervised-exercise training could be an effective strategy in managing and preventing NAFLD in children and adolescents [24]. Both aerobic and RT, at vigorous or moderate-to-vigorous intensities, with ≥60 min/sessions at a frequency of ≥3 sessions/week with the aim of improving cardiorespiratory fitness and muscular strength, had benefits on hepatic fat content reduction in young people. This data is in line with the international recommendations on physical activity for promoting health in young people, and may be useful when designing exercise training programs for improving and preventing hepatic steatosis in the paediatric population [25]. 

The exact mechanisms through which high grip strength might independently influences NAFLD have not been elucidated, but it has been hypothesized that increased muscular strength might improve insulin sensitivity and secretion, control of lipid metabolism, and increased secretion of myokines, consequently being involved in the development of NAFLD at an early age [26].

Currently, diagnosing and treating NAFLD is important in paediatric populations because metabolism-related problems may appear early in childhood and persist into adulthood, with high levels of morbidity and mortality [27]. Muscle strength measure, rather than skeletal muscle mass itself could play a moderate role on the association between anthropometric/body composition parameters and NAFLD. Grip strength and normalized grip strength (i.e., grip strength/body mass), which was selected as the primary measure of muscle strength in this study, is easy to use in both clinical and community settings and, recently, our research group proposed normalized grip strength (NGS) cut-off levels for a large sample of schoolchildren from Colombia to detect metabolic syndrome [28].

However, to the best of our knowledge, no previous studies have investigated the relationship between muscular strength and NAFLD in young populations. Thus, the aim of this study was to examine whether grip strength moderates the association between anthropometric and body composition parameters and controlled attenuation parameter (CAP), an indicator of fat deposits in the liver, in a sample of Colombian youths with excess of adiposity.

## 2. Experimental Section

### 2.1. Study Design, Setting, and Participants

The analysis involved a total of 127 children and adolescents (67% girls) aged 11–17 years from baseline analysis of the clinical trial Exercise Training and Hepatic Metabolism in Overweight/Obese Adolescent (HEPAFIT), ClinicalTrials.gov Identifier: NCT02753231, was carried out between October 2017 and January 2018. Details of background and design methods of the HEPAFIT Study had been previously published elsewhere [29]. The following inclusion criteria were adopted: primary overweight/obese status, defined according to the International Obesity Task Force (IOTF) [30], or excess of adiposity (body fat >30% by dual-energy X-ray absorptiometry (DXA)), inactivity (no participation in exercise more than once a week in the previous six months), and having at least one parent or caregiver willing to participate in the program sessions. The exclusion criteria included having a clinical diagnosis of cardiovascular disease, having type 1 or type 2 diabetes mellitus, being pregnant, using alcohol or drugs, and not having lived in Bogotá for at least one school year. Adolescents with other causes of liver disease in the paediatric population that produce elevated liver enzyme levels will be excluded. All measurements were taken on a non-regular school day. All participants were informed of the study’s goals, and written informed consent was obtained from participants and their parents or legal guardians. The study received ethical approval from the Medical Research Ethics Committee of the University of Rosario (ID CEI-ABN026-000140) and conducted in accordance with the Declaration of Helsinki.

### 2.2. Physical Fitness Parameters

Grip strength was measured using a standard adjustable handle digital handgrip dynamometer T-18 TKK SMEDLY III^®^ (Takei Scientific Instruments Co., Ltd., Niigata, Japan). Two trials were allowed for each limb and the average score recorded the peak grip strength (kg). Grip strength was normalized as grip strength per body mass, i.e., NGS (grip strength in kg)/(body mass in kg) [31]. This allowed us to be more accurate when comparing individuals with different body sizes and to focus on muscle quality rather than muscle quantity [28].

Maximum oxygen consumption (VO_2_max, mL/kg/min) was assessed by the 20 m shuttle-run test. Youths were required to run in a straight line between two lines 20 m apart, while keeping pace with a pre-recorded audio CD. We estimated the VO_2_max according to the number of laps performed Leger et al. [32]. The feasibility, reliability, and maximality of this test in adolescents have been reported elsewhere [31].

### 2.3. Anthropometric and Body Composition Measures

Anthropometric assessment included sitting height and height was measured to the nearest 0.1 cm using a portable stadiometer with a precision of 0.1 mm and a range of 0–2.50 m (Seca^®^ 206, Hamburg, Germany), and body weight was measured to the nearest 0.1 kg in light clothing and without shoes using standard digital scale (Model Tanita^®^ BC-418^®^, Tokyo, Japan). Body mass index (BMI) was calculated as weight (kg)/height (m^2^), and BMI-z score was calculated using WHO Anthro-Plus program (AnthroPlus software^®^, version 1.0.4, World Health Organization, Geneva, Switzerland, 2011). Waist circumference (WC) was measured to the nearest 0.1 cm between the lower rib margin and the iliac crest in the horizontal plane using a tape measure, with the subject standing comfortably with weight distributed evenly on both feet. Somatic maturity was captured by peak height velocity (PHV) as proposed by Mirwald et al. [33]. To indicate age at PHV, PHV was subtracted from chronological age. Waist-to-height ratio (WHtR) was calculated as the ratio of WC (in cm) to Ht (in cm). Anthropometric variables were measured by a Level 2 expert certified by the International Society for the Advancement of Kinanthropometry. The same trained investigator made all anthropometrics measurements.

Body composition parameters including percentage of body fat and visceral adipose tissue were measured using dual-energy X-ray absorptiometry (DXA) (Hologic Horizon DXA System^®^, Quirugil, Florida, MI, USA) with Discovery software, version 12.3 (Bellingham, WA, USA). Scans were performed by the same trained operator, according to the laboratory standard protocol. Exchange of each site’s calibration spine phantom confirmed the reliability of pooling results from the three scanners. All subjects were assessed for all included measures related to anthropometric and body composition in the same day.

### 2.4. Controlled Attenuation Parameter

The controlled attenuation parameter (CAP), an indicator of the deposit of fat in the liver, is the ultrasonic attenuation coefficient of the ultrasonic signals used during transient elastography examination determined with a FibroScan^®^ 502 Touch device (Echosens, Paris, France). All patients were measured with the 3.5-MHz standard “M” or “XL” probe at depth between 25 and 65 mm probe, according to the manufacturer’s specifications. The technical background and reference values in the pediatric population (1 to 18 years) have been recently described in detail [34]. As reported in the literature among youths, only liver stiffness measurements with 10 validated measurements and an interquartile range/median (IQR/M) <30% for CAP were considered reliable [35]. CAP values ≥225 dB/m defined the presence of hepatic steatosis according to Desai et al. [36]. This value shown 0.87 sensitivity, 0.83 specificity, positive predictive value 0.71, negative predictive value 0.93, and area under curve (AUC) 0.93 (95% CI 0.87–0.99). These measurements were performed in the Centre for Studies of Physical Activity Measurements (in Spanish, CEMA: Centro de Estudios en Medición de la Actividad Física), School of Medicine and Health Sciences, University of Rosario, Bogotá, Colombia.

### 2.5. Statistical Analysis

To evaluate whether or not the data were normally distributed both statistical (Kolmogorov-Smirnoff test) and graphical methods (normal probability plots) were applied. Sample size calculations were performed for the original HEPAFIT study [29]; however, as the current analysis was to evaluated the interactions between grip strength and both liver fat and anthropometry/body composition, we calculate a *post hoc* sample size calculations linear multiple regression, random model: H1 ρ^2^ = 0.5, H0 ρ^2^ = 0.1, α = 0.05, predictors outcomes = 3, *R*^2^ = 0.51, and 1 − β = 0.95. Thus, the power level to 0.90% requires 100 participants.

The descriptive statistics (i.e., mean and prevalence) on the characteristics of study participants (i.e., age in years, PHV, anthropometric and body composition parameters and CAP) were compared by gender using *t*-test or chi-square test for the continuous and categorical measures, respectively. Regression analysis was used to analyse the associations between anthropometrics and body composition parameters and the CAP in youth with obesity. The associations of the anthropometrics and body composition parameters and the CAP were analysed by linear regression using four separate models. We entered CAP as a dependent variable, anthropometrics and body composition markers as an independent variable in four separate models: Model 1: was adjusted for sex and peak height velocity (years); Model 2 was adjusted for model 1 + maximal oxygen uptake; Model 3: was adjusted for model 1 + handgrip strength/weight; and Model 4: was adjusted for model 1 + model 2 + model 3.

Finally, regression and moderation analyses were conducted using the PROCESS macro 2.16 in IBM SPSS (IBM SPSS Statistics for Windows, Version 22.0, Armonk, NY, USA). PROCESS utilizes ordinary least squares regression analysis when predicting continuous variables (CAP in the current study) and a bootstrap method (with 5000 bootstrapped samples) to estimate the moderated effects [37]. Bootstrapping is the statistical method of random resampling, with replacement, from the sample distribution to create an approximate comparison distribution. This approximate distribution is used for hypothesis testing, rather than testing against a known distribution (e.g., *z*-distribution, *t*-distribution). To probe significant interactions, simple slope analysis at low (−1 SD), average (mean), and high (+1 SD) levels of the moderator was used with the Johnson-Neyman technique. The Johnson-Neyman technique was applied to the conditional model in order to determine a threshold of significance [38]. This parameter is an analysis of covariance (ANCOVA) where the relationship between anthropometric and body composition parameters are assumed to be linear but nonparallel at varying degrees of the moderator (NGS). A region of significance is then identified by applying fixed values of NGS, across the range of sample data, to the regression equation. Bias-corrected and accelerated confidence intervals were used for hypothesis testing, meaning that the confidence intervals adjusted for skewness and any over- or under-estimation of the population parameter. The Johnson-Neyman technique thus provides greater resolution for clarifying interactions than traditional techniques as lineal regression or mediation analysis.

In the context of the current study, the technique highlights specific NGS cut-points in which the significant relationship between anthropometric and body composition parameters and the CAP disappears, as well as how that relationship varies based on the changes of NGS.

## 3. Results

Table 1 describes the characteristics of the study sample by sex. Among the participants, 68 youths (53.5%) presented NAFLD. The mean values of WC, WHtR, VAT, VO_2_max, and CAP were significantly higher in boys than in girls, *p* < 0.01. In contrast, age, PHV and NAFLD prevalence were significantly higher in girls than boys, *p* < 0.01. No significant differences were observed between boys and girls for the remaining variables.

Associations between anthropometrics and body composition parameters and CAP in obese adolescents are shown in Table 2. In all the models, the anthropometric and body composition parameters were positively associated with CAP (range β = 0.423 to 0.580), slightly reduced after being adjusted for NGS (i.e., model 3).

**Figure 1:**
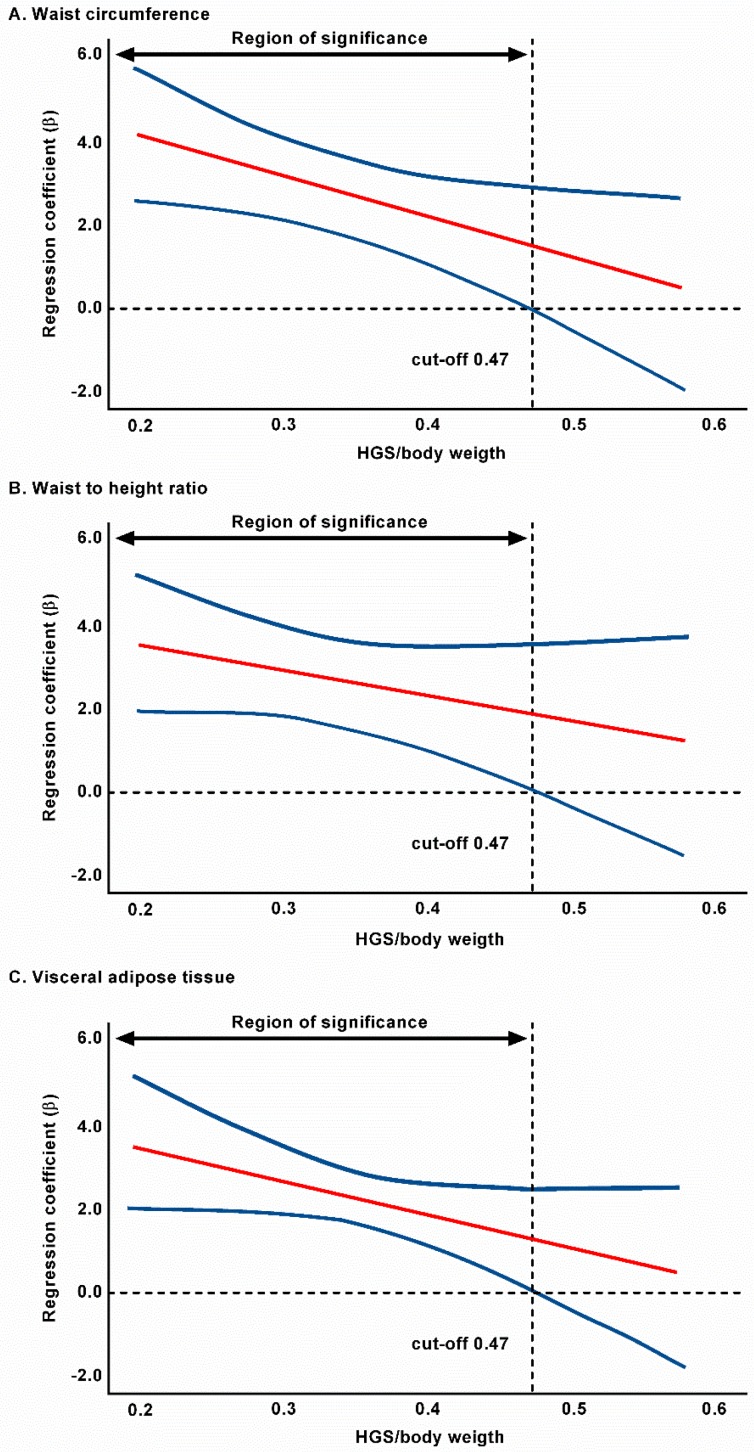
Regression slope estimate and 95% confidence interval for the relationship between anthropometric and body composition parameters and controlled attenuation (**A**), waist circumference; (**B**), waist to height ratio; and (**C**), visceral adipose tissue, as a function of HGS/weight, (moderator), based on Johnson-Neyman results. HGS, handgrip strength.

Finally, the regression slope estimated for the relationship between anthropometric and body composition parameters and CAP is shown in Table 3. The Johnson-Neyman technique revealed a significant inverse relationship between WC, WHtR, VAT, and CAP when grip strength/weight was above 0.475 (8.1% of the sample), 0.469 (8.9% of the sample), and 0.470 (8.5% of the sample), respectively. The direction of this relationship became negative as NGS increased (Figure 1).

## 4. Discussion

This is the first study to assess whether grip strength moderates the association between anthropometric and body composition parameters and CAP in a sample of children and adolescents with excess of adiposity. Our results demonstrated that NGS is indeed a moderator of the association between WC, WHtR and VAT, and CAP in youths. Additionally, the current study showed the prevalence of NAFLD among an urban Colombian sample population, which indicated that 59.5% of boys and 50.5% of girls suffered from the disease.

To the best of the researchers’ knowledge, no other study has considered grip strength as a moderator of the relationship between anthropometric/body composition parameters and fatty liver. In this line, our findings revealed a significant inverse relationship between WC, WHtR, VAT, and CAP when HGS was above 0.475, 0.469, and 0.470, respectively, supporting the fact that grip strength moderates these associations in youths with excess of adiposity. Consistent with these findings, increased handgrip strength was found to be independently associated with a lower prevalence of NAFLD in a large-scale adult sample [39]. In this population, previous studies have also reported associations between muscle mass and NAFLD [20]. In a study of Korean subjects, skeletal muscle mass inversely correlated with the fatty liver index, suggesting that higher skeletal muscle mass may play a beneficial role in preventing NAFLD [9]. Furthermore, Kim et al. [6] showed that a low skeletal muscle index was independently associated with a risk of NAFLD using the fatty liver index. It can be assumed that muscle quality (strength per unit of muscle size or mass) affect fatty liver by changing WC, WHtR and VAT size, which is an indication of fat accumulation. Therefore, this study, in line with studies mentioned above, supports NGS as a potentially useful moderator of the association between anthropometric and body-composition parameters and NAFLD. It should be hypothesized that increased muscular strength might generate a higher basal metabolic rate and greater energy expenditure that may result in reduced visceral adipose tissue and less fatty deposits in the liver.

On the other hand, we found that cardiorespiratory fitness by maximal oxygen uptake is not a moderator in the association between anthropometric/body composition parameters and fatty liver. To our knowledge, no previous studies have investigated specially the effect of cardiorespiratory fitness on NAFLD. Perseguin et al. [21] reported that a higher level of habitual physical activity was associated with a lower intrahepatic fat content and suggested that this relationship may be due to the effect of exercise per se. Moreover, regular exercise was associated with a reduced risk for having NAFLD in patients with NAFLD, and this relationship was also independent of obesity [20]. However, it is important to highlight that in these studies physical activity was assessed by a self-reported questionnaires. Additionally, the contradictory results observed may be explained by the differences in sample characteristics such as age range and ethnicity.

This study has several strengths. Firstly, we used liver CAP, a highly sensitive, non-invasive, and accurate technique for assessing liver fat accumulation. Finally, to the best of our knowledge, this is the first study to investigate the role grip strength plays in the association between anthropometric and body composition parameters and liver stiffness in a sample of youths, as all previous studies have involved cohorts of adults. For that reason, here we provide the rationale for proceeding with prospective studies to confirm the positive influence of muscular strength on CAP during children and adolescence.

On the other hand, a potential limitation of our study is that our findings could not establish casual relationships between anthropometric and body composition parameters, CAP, and grip strength, because of the cross-sectional design. Thus, future longitudinal studies are required to elucidate the role of muscular strength in moderating this complex relationship. Other limitations of this study include lack of a biopsy, the most accurate method of assessing the extent of liver damage in NAFLD, which could not be performed on the schoolchildren. Although liver biopsies are the gold-standard method, this procedure is not recommendable for all subjects due to limitations including its invasiveness, cost, and potentially life-threatening complications. It should be also noted that a major threat against internal validity of this study might be genetic basis, which are not controlled for and thus may constitute unobserved heterogeneity. Additionally, other factors that are not controlled for such as youth’s parents’ socio-economic status and health care access might confound the relationship between the variables under examination. Similarly, other factors that may result in confounding variables including behavioural habits, especially those related to nutrition, physical activity, substance use (e.g., smoking), and sleep in youth have not been considered.

## 5. Conclusions

In conclusion, this study demonstrates that handgrip strength moderates the associations between anthropometric and body composition parameters including WC, WHtR, and VAT, and CAP in children and adolescents with excess of adiposity. These findings are clinically significant because NAFLD has the potential to progress to cirrhosis and a need for liver transplant in childhood and early adulthood. Given that our study shows grip strength moderates the relationship between anthropometric and body composition indicators and NAFLD as captured by CAP, it may be possible that screening for grip strength and other muscular strength and emphasizing importance of physical fitness during youth may help improve health-related outcomes for children with excess adiposity. More studies with appropriate research designs and data that can enable causal inference are needed.

## Figures and Tables

**Table 1:** Descriptive characteristics of participants by sex.

Characteristics	Boys (*n* = 42)	Girls (*n* = 85)	*p*-Value
Chronological age, years	12.9 (1.2)	13.7 (1.7)	0.003
Age of PHV, years	12.3 (0.6)	14.4 (0.6)	0.001
Anthropometric parameters			
Body mass index, kg/m^2^	24.2 (2.5)	23.5 (4.1)	0.359
Body mass index, *z*-score	1.73 (0.64)	1.39 (0.85)	0.013
Overweight + obese prevalence (%) *	41.1	55.9	0.066
BF > 30% by DXA prevalence (%) *	97.6	100	0.997
Waist circumference, cm	79.4 (6.8)	74.6 (8.4)	0.009
Waist-to-height ratio	0.505 (0.039)	0.480 (0.052)	0.009
Body composition parameter			
BF% by DXA	40.8 (4.1)	38.0 (4.6)	0.001
Visceral adipose tissue (cm^3^)	382.9 (82.4)	323.1 (108.0)	0.003
Vibration controlled transient elastography			
Controlled attenuation parameter, dB/m	245.8 (41.9)	216.2 (40.9)	<0.001
Liver stiffness, kPa	3.9 (0.7)	4.0 (3.1)	0.850
NAFLD prevalence, *n* (%)	25 (59.5)	43 (50.5)	0.010
Physical fitness parameters			
Handgrip strength (kg)	21.6 (6.4)	20.7 (4.7)	0.376
Handgrip strength, (kg)/Weight, (kg)	0.37 (0.07)	0.36 (0.07)	0.485
VO_2_max (mL/kg/min)	39.4 (3.8)	37.2 (3.1)	0.001
Shuttles (total count)	21.3 (15.9)	18.1 (9.0)	0.268
Stage (last completed)	3.2 (1.9)	2.9 (1.1)	0.161
Running speed at last completed shuttle (km∙h^−1^)	9.6 (0.9)	9.4 (0.6)	0.291

Data are reported as mean values (standard deviation, SD) or percentages. Significant between-sex differences (*t*-tests or * chi-squared test *X^2^*). *p* values of 0.05 are considered statistically significant. BF%: body fat percentage; *z*-BMI: *z*-score of body mass index; DXA: dual energy X-ray absorptiometry; VO_2_max: maximal oxygen uptake; PHV: peak height velocity; NAFLD: Non-alcoholic fatty liver disease. Equations to estimate VO_2_max in boys and girls = 31.025 + 3.238 × (S × (3.248 × (A + 0.1536 × (S × A), where A is age and S is final speed (S = 8 + 0.5 × last stage completed).

**Table 2:** Associations between anthropometrics and body composition parameters and the controlled attenuation parameter in youths with obesity.

Parameter	β (Standardized)	*p*-Value
Waist circumference (cm)		
Model 1	0.564	<0.001
Model 2	0.574	<0.001
Model 3	0.510	<0.001
Model 4	0.526	<0.001
Waist-to-height ratio		
Model 1	0.550	<0.001
Model 2	0.558	<0.001
Model 3	0.484	<0.001
Model 4	0.500	<0.001
Fat mass (kg)		
Model 1	0.478	<0.001
Model 2	0.481	<0.001
Model 3	0.423	<0.001
Model 4	0.435	<0.001
Visceral adipose tissue (cm^3^)		
Model 1	0.580	<0.001
Model 2	0.576	<0.001
Model 3	0.535	<0.001
Model 4	0.537	<0.001

Model 1: analyses adjusted for sex and peak height velocity (years). Model 2: analyses adjusted for model 1 + maximal oxygen uptake. Model 3: analyses adjusted for model 1 + handgrip strength/weight. Model 4: analyses adjusted for model 1 + model 2 + model 3. *p* values of 0.05 are considered statistically significant.

**Table 3:** Regression slope estimates for the relationship between anthropometrics and body composition and the controlled attenuation parameter with grip strength as moderator based on Johnson-Neyman results.

Physical Fitness Parameter	WC		WHtR		Fat Mass		Visceral Adipose Tissue	
	Moderator ^#^	*p*-Value	Moderator ^#^	*p*-Value	Moderator ^#^	*p*-Value	Moderator ^#^	*p*-Value
VO_2_max, mL/kg/min	No interaction	0.812	No interaction	0.485	No interaction	0.291	No interaction	0.760
Grip strength/weight	0.475 *	0.027	0.469 *	0.037	No interaction	0.318	0.470*	0.019

* Interaction; **^#^** Moderator value defining Johnson-Neyman significance region. Analysis adjusted by sex, peak height velocity (years) and maximal oxygen uptake or NGS according to dependent variable included in the model. *p* values of 0.05 are considered statistically significant. VO_2_max: maximal oxygen uptake. WC: Waist circumference. WHtR: waist-to-height ratio.
